# Tuberculosis-Associated Immune Reconstruction Inflammatory Syndrome (TB-IRIS) in HIV-Infected Patients: Report of Two Cases and the Literature Overview

**DOI:** 10.1155/2013/323208

**Published:** 2013-04-18

**Authors:** Klaudija Viskovic, Josip Begovac

**Affiliations:** ^1^Department of Radiology and Ultrasound, University Hospital for Infectious Diseases, Mirogojska Cesta 8, 10000 Zagreb, Croatia; ^2^Center for HIV/AIDS Infection, University Hospital for Infectious Diseases and School of Medicine, University of Zagreb, Croatia

## Abstract

We describe two HIV-infected patients with tuberculosis-associated immune reconstruction inflammatory syndrome (TB-IRIS): one with “paradoxical” IRIS and the other with “unmasking” IRIS. TB-IRIS in HIV-infected subjects is an exacerbation of the symptoms, signs, or radiological manifestations of a pathogenic antigen, related to recovery of the immune system after immunosuppression. We focused on the radiological characteristics of TB-IRIS and the briefly literature review on this syndrome.

## 1. Introduction

Tuberculosis- (TB-) associated immune reconstruction inflammatory syndrome (TB-IRIS) is emerging as an important early complication of combination antiretroviral therapy (CART) in patients with HIV infection all over the world. In Europe, and United States studies the reported incidence of TB-IRIS was from 11% to 45% [1–4]. The frequency of TB-IRIS among Croatian patients in the study of Puljiz and colleagues in 2006 was 40.7% [5].

IRIS is a condition that results from rapid restoration of pathogen-specific immune responses to opportunistic infections, causing either the deterioration of a treated infection or the new presentation of a previously subclinical infection [6, 7]. There is no diagnostic test for IRIS, and a confirmation of the disease relies heavily upon case definitions incorporating clinical and laboratory data [7]. Case definitions for IRIS were published by French and collaborators in 2004 and by Shelburne and colleagues in 2006 [8, 9]. A case definition specific for TB-IRIS was also suggested by Colebunders et al. in 2006 [10]. However, clinical management and research on IRIS were hindered by the lack of consensus case definitions and definitions that are specific to particular opportunistic infections [7]. To address this issue, in resource-limited settings, an international meeting of researches working in this field was convened in Kampala, Uganda in November 2006, and the international network for the study of HIV-associated IRIS (INSHI) was formed [7]. INSHI published criteria for “paradoxical” TB-IRIS and “unmasking” TB-IRIS diagnosis in 2008 aimed for use in settings in which laboratory infrastructure is often limited [7]. 

The phenomenon of “paradoxical” reaction during the treatment of TB in which existing disease may worsen or new lesions appear has been recognized for many years, before the association with HIV coinfection [11]. Most reported cases have complicated the treatment of lymph node or cerebral disease, with enlargement of nodes seen in approximately 30% in one large series [12, 13]. In the past few years an increase in the frequency and severity of paradoxical reactions in association with HIV coinfection has been reported both in patients receiving CART and those not on CART [1].

A diagnosis of  “paradoxical” IRIS required both a diagnosis of TB and an initial response to antituberculous therapy (ATT) together with at least one of four major or two of three minor clinical criteria and exclusion of alternative explanations (Table 1) [7, 14].

The INSHI definition of CART-associated “unmasking” TB requires three criteria: not receiving TB treatment at CART initiation; diagnosis of active TB after CART initiation; fulfilling World Health Organization (WHO) diagnostic criteria for TB [7]. Additional criteria required for “unmasking” TB-IRIS are presentation within three months of CART initiation and either “heightened intensity of clinical manifestations or development of a paradoxical reaction once on TB treatment (Table 2) [7, 14, 15].

In this case report and overview of the literature, we present the disease spectrum of “paradoxical” and “unmasking” TB-IRIS in two patients with complete resolution of disease. 

## 2. Patients and Methods

Both patients received medical care at the University Hospital for Infectious Diseases (UHID) in Zagreb, Croatia, which provides comprehensive care of all HIV-infected patients in Croatia. Croatia has a low-level HIV epidemic, and there were 792 registered HIV/AIDS cases at the end of 2009, according to the HIV/AIDS Register of the Croatian National Institute of Public Health [16, 17]. The definition of TB-IRIS in our patients required a case definition criterion for “paradoxical” TB-IRIS that consists of three components as displayed in Table 1 or fulfilling criteria of “unmasking” TB-IRIS displayed in Table 2 [7]. Informed consent was obtained from both patients granting us the permission to publish the details of their medical presentation and management.

## 3. Case Presentations 

### 3.1. Case 1 Patient with “Paradoxical” TB-IRIS

A 53-year-old Caucasian female was admitted to UHID in December 2006 with a three-week history of fever and cough. HIV-1 infection was diagnosed in 2004, and CART was prescribed in April 2004. However, the patient stopped taking CART in December 2005.

At admission, chest radiographs showed patchy medial lobe infiltrate and massive right hilar adenopathy. Native and postcontrast thoracic computed tomography (CT) scan confirmed large right side mediastinal and pulmonary lymph nodes. High resolution CT scan of the right lung showed peripheral, poorly defined, small (2–4 mm diameter) centrilobular nodules and branching linear opacities of similar caliber originating from a single stalk (the “tree-in-bud” pattern) [18]. No cavities were detected. Sputum cultures grew *Mycobacterium tuberculosis* (*M. tuberculosis)* sensitive to all ATT drugs. 

**Figure 1:**
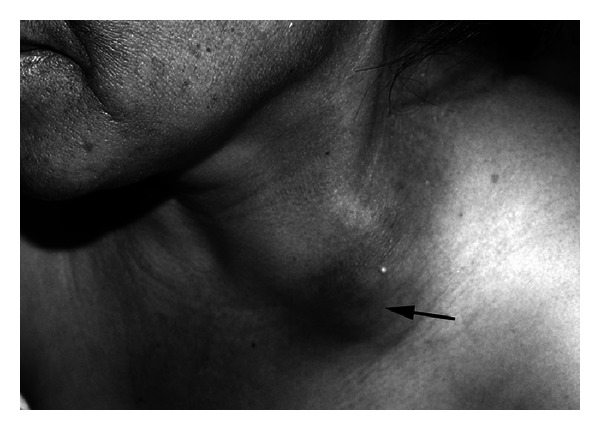
Enlarged cervical lymph nodes in a patient (Case no. 1) with “paradoxical” tuberculosis- (TB-) associated immune reconstruction inflammatory syndrome (TB-IRIS) (arrow).

Standard ATT (isoniazid, rifampin, pyrazinamide, and ethambutol) was initiated in December 2006 and was given for 8 weeks. After that, isoniazid and rifampin were given for the period of 12 months. CART (stavudine, lamivudine, and efavirenz) was given 15 days after initiation of ATT. In February 2007, three months after initiation of ATT, two “cold” abscesses of thoracic wall were noticed and confirmed by ultrasound (US) and CT imaging. We performed initial aspiration biopsy to establish the diagnosis and to exclude malignancy [19]. Eventually, surgical incision and drainage was done. Pulmonary infiltrates at that time were completely resolved. In August 2007, the patient noted enlarged cervical lymph nodes. On physical examination performed at that time, we found enlarged lymph nodes in the left anterior cervical chain and left supraclavicular region (Figure 1). The involved areas were minimally tender with no warmth or erythema. The largest lymph node was approximately 3 × 4 cm, and the consistency of the lymph nodes varied from firm to fluctuant. CT and US scans of the neck, performed in August 2007, revealed large left side lymphadenopathy along the anterior cervical chain and in the supraclavicular region. Most of the lymph nodes were noted to be necrotic. A US-guided fine needle aspiration of two largest, necrotic cervical nodes was performed. About 70 mL of necrotic, purulent material was removed. Cytological examination showed granulomatous inflammation with caseous necrosis. Acid-fast bacilli (AFB) were seen, but cultures did not grow *M. tuberculosis*. IRIS was treated with steroids (methylprednisolone) for 10 months from January to November 2007. 

**Figure 2:**
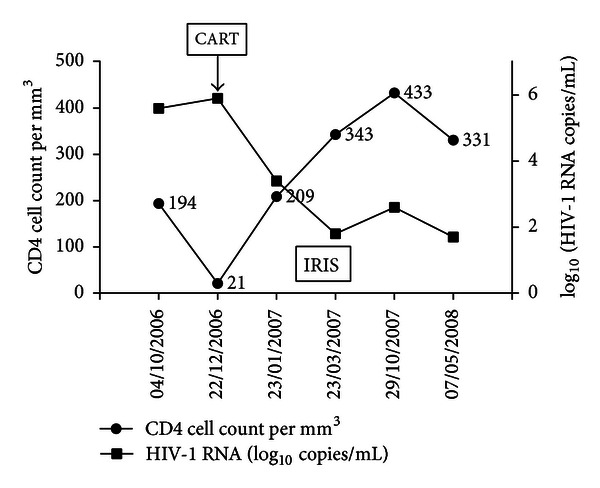
The CD4 cell count and viral load (HIV RNA copies/mL) over time in a patient with “paradoxical” tuberculosis- (TB-) associated immune reconstruction inflammatory syndrome (IRIS). CART: combination antiretroviral therapy.

The CD4 cell count and viral load (HIV RNA copies/mL) over time are presented in Figure 2.

The patient's health condition slowly improved over the next 12 months, and in August 2008, the neck lymphadenopathy and thoracic wall abscesses had resolved with only scar tissue remaining at the site of previous surgical incision.

### 3.2. Case 2 Patient with “Unmasking” TB-IRIS

A 40-year-old Caucasian male came to a routine followup at the HIV/AIDS Outpatient Center at the University Hospital for Infectious Diseases (UHID) in Zagreb in February 2008. On physical examination, a painless mass in the left inguinal region was noted. The skin overlying the region was not erythematous, and the patient had no fever. He stated having intermittent low-grade fever and a minor swelling in the left groin since October 2007. 

He was known to be HIV infected after a tonsillectomy performed in May 2007. Pathohistological examination of the tonsils revealed chronic granulomatous infection. At that time, he had a reactive purified protein derivative (PPD) test which was positive (measured 18 × 20 mm). The chest X-ray was normal. CART was commenced with abacavir, lamivudine, and efavirenz in July 2007. No TB prophylaxis was given.

In a car accident in August 2007, he had lumbar region contusion, and eventually the lumbar spine CT scan was performed to exclude bone fractures. The CT scan showed no evidence of traumatic or other bone lesions of lumbar spine vertebrae. The paravertebral spaces were normal.

**Figure 3:**
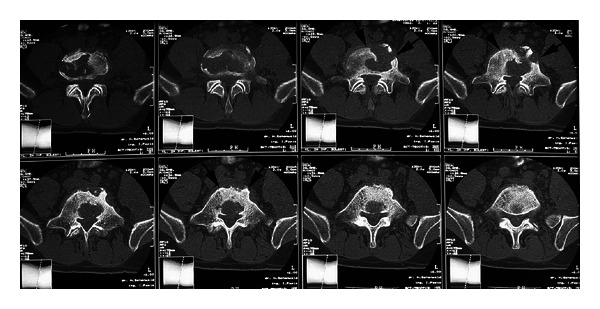
CT scans demonstrated permeative bone destruction of the lower end plate of the fourth lumbar vertebra and the upper end plate of the fifth lumbar vertebra, consistent with spondylodiscitis in a patient with “unmasking” tuberculosis- (TB-) associated immune reconstruction inflammatory syndrome (TB-IRIS) (arrows) (Case no. 2).

**Figure 4:**
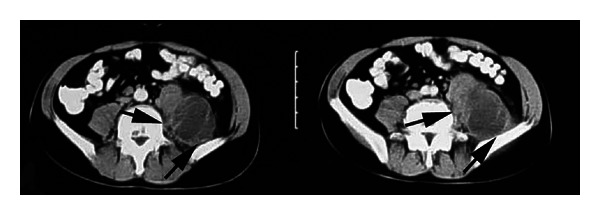
Axial, contrast-enhanced abdominal computed tomography (CT) scans showed huge abscess in a left psoas muscle (arrows) in a patient with “unmasking” tuberculosis- (TB-) associated immune reconstruction inflammatory syndrome (TB-IRIS) (Case no. 2).

After the above mentioned visit in February 2008, the patient was admitted to UHID. Abdominal US revealed hypoechoic mass in the left psoas muscle extending in the left inguinal region above the inguinal ligament. Axial, contrast-enhanced lumbar spine and abdominal CT scans demonstrated permeative bone destruction of the lower end plate of the fourth lumbar vertebra and the upper end plate of the fifth lumbar vertebra, consistent with spondylodiscitis and a huge left psoas abscess (about 35 × 10 × 8 cm) (Figures 3 and 4).

**Figure 5:**
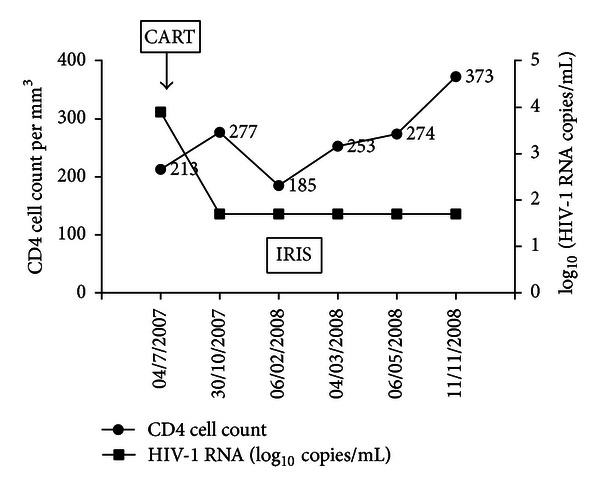
The blood CD4 cell count and HIV-1 RNA response to CART over time in a patient with “unmasking” tuberculosis- (TB-) associated immune reconstruction inflammatory syndrome (TB-IRIS). CART: combination antiretroviral therapy.

A lumbar puncture was performed and was unremarkable. The chest radiograph was normal. The blood CD4 cell count was 185/mm^3^, and the HIV plasma viral load was <50 copies per mL. We present the blood CD4 cell count and HIV-1 RNA response to CART over time in Figure 5.

US-guided aspiration of psoas abscess was performed, which revealed purulent material. AFB staining was positive, and subsequently *M. tuberculosis* was cultured. It was sensitive to all major AT drugs. The patient was subsequently referred to the Department of Surgery at the University Traumatology Clinic, Zagreb, where surgical drainage via an extra peritoneal approach was performed. The abscess was completely drained, and 1400 mL of pus was evacuated. 

Treatment with rifampin, ethambutol, isoniazid and pyrazinamide was initiated in February 2008 and was given for 8 weeks. After that isoniazid, and rifampin were given for the period of 12 months. 

**Figure 6:**
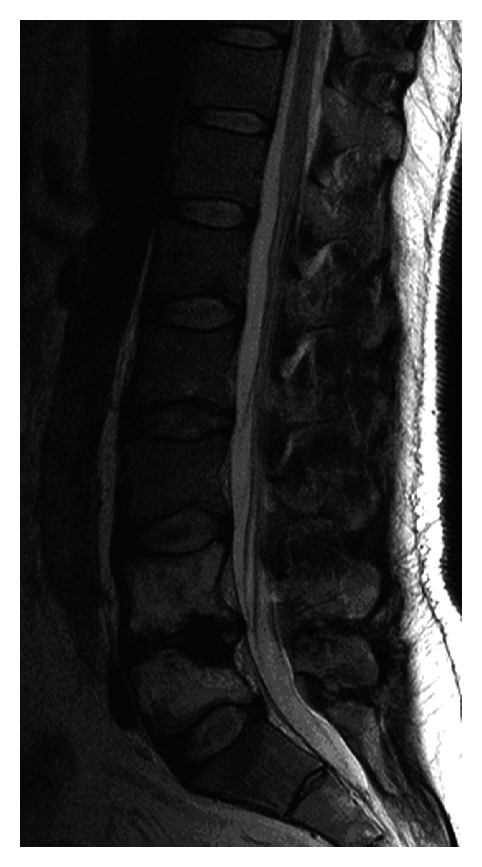
A follow-up magnetic resonance imaging (MRI) examination of the spine performed in September 2008 showed high signal intensity of L4 and L5 lumbar vertebral bodies in T2-weighted images (WIs) as signs of healing, with loss of height from infective destruction. There was no spinal canal stenosis.

Over the next nine month,s the patient had several further outpatient follow-up visits. A follow-up magnetic resonance imaging (MRI) examination of the spine was performed in September 2008. It showed high signal intensity of L4 and L5 lumbar vertebral bodies in T1- and T2-weighted images (WIs) as signs of healing, with loss of height from infective destruction. There was no spinal canal stenosis (Figure 6). During the visit in November 2008, all signs and symptoms of TB resolved. 

## 4. Discussion 

The patient with “paradoxical” form of TB-IRIS fulfilled the INSHI criteria: the diagnosis of TB was made before reinitiation of CART and fulfilled WHO criteria for diagnosis of smear-positive pulmonary TB and extra pulmonary TB [7]. The onset of TB-IRIS manifestation was within 3 months of CART initiation. The pulmonary infiltrate resolved after two months of ATT, but at the same time thoracic wall cold abscesses appeared, followed by enlarged, necrotic lymph nodes at the supraclavicular region and neck. We excluded possible alternative explanations for clinical deterioration: poor adherence to ATT because the patient was hospitalized and carefully monitored, and the presence of another opportunistic infection or neoplasm was excluded by different diagnostic methods. Drug resistance was excluded by testing with culture techniques [20].

The patient with spondylodiscitis and psoas abscess also fulfilled the INSHI [7] criteria for “unmasking” TB-IRIS. However, there is some uncertainty about the precise time point of the onset of TB symptoms in relation to CART initiation. Our patient recalled the first symptoms of TB infection about three months after CART initiation. However, CT scans of lumbar spine and abdomen were performed on his follow-up visit seven months after CART initiation and showed signs of spondylodiscitis and psoas abscess. Because of a car accident, a CT scan of lumbar vertebrae was performed one month after CART initiation. It was unremarkable; no signs of bone destruction or trauma were found. The destruction of lumbar spine vertebrae and psoas abscess gradually developed in the course of seven months. The marked inflammation that resembled a formation of large psoas abscess supports the diagnosis of IRIS.

At the time when the patient was found to be HIV infected, the PPD test was positive. Latent TB infection (LTBI) is defined by a positive *M. tuberculosis*-specific immune response in the PPD or an interferon-gamma release assays (IGRAs) in the absence of active TB. A positive PPD test indicates an immunological memory to previous or ongoing contact with *M. tuberculosis* and may be found also in patients who were BCG vaccinated or who had contact with atypical mycobacteria [21]. Our patient was BCG vaccinated and we did not decide to give TB preventive chemotherapy. Analyzing our decision in retrospect, it is possible that we might have prevented the development of active TB by introducing preventive chemotherapy. However, preventive chemotherapy has no effect on the overall mortality [22]. The development of drug resistance to *M. tuberculosis* is also a concern when preventive therapy is given.

The majority of patients with “unmasking” IRIS had a pulmonary form of TB [23]. Only a few cases of TB spondylodiscitis without pulmonary findings have been reported [15, 24]. The lumbar and lower thoracic regions are most commonly affected in spinal TB [25]. A high index of clinical suspicion and an accurate history of possible TB exposure are essential for the diagnosis of skeletal TB in HIV-infected patients. Spinal TB has no specific diagnostic characteristics or features on radiological imaging and manifests in 2 distinct forms: spondylodiscitis (classic) and spondylitis without disc involvement (atypical) [26]. Involvement of contiguous vertebrae is common [26]. Paraspinal abscesses can develop in more than 50% of spinal TB cases, and the identification of psoas abscess needs a prompt evaluation of the spine as a primary source of infection [26, 27]. Microscopy and culture of infected material are recommended for the diagnosis of spinal TB [25]. Needle aspiration and biopsy, preferably CT-guided, are advocated and considered both sensitive and specific [28].

“Paradoxical” reactions generally occur within 1 to 3 months of initiation of treatment [29]. Why “paradoxical” reactions to drug therapy should occur only in some individuals is unclear. It is likely to be due to a complex interplay of the host immune responses, tubercle bacilli virulence, antigen load, the site of infection, and the effects of chemotherapy [30]. The massive delivery of membrane antigens after the initiation of ATT has been advocated as the cause of “paradoxical” responses in immunoincompetent patients [30]. Another possible mechanism is immunologic restoration after antiretroviral therapy and recovery of specific responses to certain antigens [1].

The most frequent risk factors for the development of IRIS found in the majority of studies were starting CART close to the treatment of opportunistic infection, a low CD4 cell count, and a high viral load [31]. In low-income countries, in districts where TB and CART services are not integrated and delivered in different localities, delays in starting CART were largely attributable to the prolonged time between TB diagnosis in the TB clinic and patients access to the CART in the HIV clinic [32, 33]. 

The treatment of suppurative lymphadenitis and cold abscesses includes drainage of the purulent material. Spontaneous drainage may result in scar formation; hence, repeated punctures or surgical drainage is preferred. We performed US-guided puncture and evacuation of necrotic neck lymph nodes in our patient. The TB abscess of the chest wall was at one time point incised and drained. 

According to our experience and the literature search, we recommend the following general principles of treatment based on existing reports, trials, and expert opinions [14, 34–39].

- Clinicians should become familiar with the case definitions for TB-IRIS and use specific definitions developed for resource-limited settings. Clinicians should consider alternative diagnoses in patients with suspected TB-IRIS, especially if the severity of the condition warrants administering corticosteroids.
- Patients should continue antituberculous therapy without change unless there is a reason to suspect that the current regimen is inadequate.
- In nearly all cases, patient with TB-IRIS should remain on antiretroviral therapy. Certain circumstances, however, may require temporary interruption of antiretroviral therapy, such as life-threatening central nervous system complications of IRIS.
- We recommend performing incision and drainage (with stains and cultures) for localized abscesses, as it was in the case of the psoas abscess in our patient.
- Although criteria for initiating corticosteroids remain poorly defined, we recommend administering prednisone for TB-IRIS cases when patients have persistent fever that affects everyday activities.
- The dosing and duration of corticosteroids should be tailored to individual patient circumstances.
- The patient receiving corticosteroids for TB-IRIS should undergo careful monitoring for response to corticosteroid therapy and monitoring for the development of possible concurrent infections or other corticosteroid side effects. Prophylaxis for opportunistic infections) should also be given.
- If the patient's clinical condition does not improve, the clinician should consider an alternative diagnosis and possible termination of corticosteroids, since administering corticosteroids to a patient with a non tuberculous opportunistic infection or disease could result in further clinical deterioration.

## Figures and Tables

**Table 1:** Case definition for “paradoxical” tuberculosis-associated immune reconstruction inflammatory syndrome (TB-IRIS) for use in resource-limited settings [7].

Three components to this case definition:	
(1) *Antecedent requirements *(*both of the following requirements must be met*):	

(1) Diagnosis of TB	Made before starting of combination antiretroviral therapy (CART) and should fulfill WHO criteria
(2) Initial response to TB treatment	The patient's condition should have stabilized or improved on appropriate TB treatment before CART initiation

(2) *Clinical criteria *	

The onset of TB-associated IRIS manifestations should be within 3 months of CART initiation, reinitiation, or regimen change because of treatment failure	

Of the following, at least one major criterion or two minor clinical criteria are required:	

Major criteria	

	New or enlarging lymph nodes, cold abscesses, or other tissue involvement
	New or worsening radiological features of TB
	New or worsening central nervous system CNS TB
	New or worsening serositis

Minor criteria	

	New or worsening constitutional symptoms such as fever, night sweats, or weight loss
	New or worsening respiratory symptoms as cough, dyspnea, or stridor
	New or worsening abdominal pain accompanied by peritonitis, hepatomegaly, splenomegaly, or abdominal adenopathy

(3) *Excluded alternative explanations for clinical deterioration *	

	Failure of TB treatment because of TB drug resistance
	Poor adherence to TB treatment
	Another opportunistic infection or neoplasm
	Drug toxicity or reaction

**Table 2:** Provisional case definition for “unmasking” tuberculosis-associated immune reconstruction inflammatory syndrome (TB-IRIS) in resource-limited settings [7].

“Unmasking” TB-IRIS (provisional) The following could suggest a diagnosis of “unmasking” TB-associated IRIS:	
Patient is not receiving treatment for TB when CART is initiated and then presents with active TB within 3 months of starting CART	

And one of the following criteria must be met:	

	(1) Heightened intensity of clinical manifestations, particularly if there is evidence of a marked inflammatory component to the presentation. Examples include TB lymphadenitis or TB abscesses with prominent acute inflammatory features, presentation with pulmonary TB that is complicated by respiratory failure due to ARDS, and those who present with a marked systemic inflammatory distress syndrome related to TB
	(2) Once established on TB treatment, a clinical course that is complicated by a paradoxical reaction
